# Ultra-processed food consumption is associated with variations in daily routines in elementary schoolchildren during the COVID-19 pandemic in Chile

**DOI:** 10.1017/S1368980023001593

**Published:** 2023-10

**Authors:** Gabriela Fretes, Camila Corvalán, Christina D Economos, Norbert LW Wilson, Sean B Cash

**Affiliations:** 1 International Food Policy Research Institute (IFPRI), 1201 Eye St NW, Washington, DC 20005, USA; 2 Instituto de Nutrición y Tecnología de los Alimentos (INTA), Universidad de Chile, Avda. Macul 5524, Santiago 7830489, Chile; 3 Friedman School of Nutrition Science and Policy, Tufts University, 150 Harrison Avenue, Boston, MA 02111, USA; 4 Duke Divinity School, Sanford School of Public Policy, and World Food Policy Center, Duke University, 407 Chapel Drive, Durham, NC, 27708-0968, USA

**Keywords:** COVID-19, Ultra-processed food, Schoolchildren, Routine, School environment, Structured days hypothesis

## Abstract

**Objective:**

To assess the association between child ultra-processed food (UPF) consumption and home-school learning environment characteristics during school closures due to the COVID-19 pandemic in schoolchildren with low- and middle income in Chile.

**Design:**

Cross-sectional. UPF consumption was collected using the Nova screener. We apply the structured days hypothesis (SDH) to assess home-school learning environment characteristics with three constructs that summarised school preparedness for online teaching and learning, school closure difficulties for caregivers and child routine. We explored associations between child UPF consumption and home-school environment characteristics using multivariate linear regression analyses after controlling for child demographic and school characteristics.

**Setting:**

Low- and middle-income neighbourhoods in southeastern Santiago, Chile.

**Participants:**

Children from the Food Environment Chilean Cohort (*n* 428, 8–10 years old).

**Results:**

Based on the Nova score, child mean consumption of UPF was 4·3 (sd 1·9) groups. We found a statistically significant negative association between child routine for eating, play and study and child UPF consumption when we adjusted for child sociodemographic (model 1: *β* = –0·19, (95 % CI –0·40, 0·02)) and school characteristics (model 2: *β* = –0·20, (95 % CI –0·41, 0·00)). Associations between school preparedness for online teaching or school closure difficulties and UPF were not statistically significant.

**Conclusions:**

Variations in child routines during the COVID-19 pandemic were negatively associated with UPF intake in schoolchildren with low- and middle income. Our findings are consistent with the SDH, suggesting the school environment helps regulate eating behaviours. Future research should evaluate what happens when children return to in-person classes at school.

In response to the COVID-19 outbreak in March 2020, the Chilean government implemented compulsory strict confinement in most regions of the country and school closures nationally as measures to slow the transmission of the SARS-CoV-2, the virus that causes COVID-19^(1)^. Recent studies have documented that school closures resulted in changes in the instructional modalities^(2,3)^, modifications to school meals delivery mechanisms^(4,5)^ and disruptions in children’s daily routines^(6,7)^. Furthermore, prolonged school closures and home confinement due to the pandemic might have had nutritional, educational and health consequences for school children^(8,9)^.

School days are considered an example of an ‘ever-present structured environment with purposive, segmented, restrictive and compulsory components’^(10)^. According to the structured days hypothesis (SDH), the structured environment of school weekdays may help protect children by regulating obesogenic behaviours such as unhealthy eating^(10)^. Research suggests that when children are out of school (i.e. weekends, summer vacations), they navigate a more autonomous environment that offers more choice and less structure, which negatively influence schoolchildren’s physical health, especially in low-income households^(11)^. Although the school environment is not without choice, if it is regulated, it can improve the availability of more healthful options to consume throughout the day^(12)^. The COVID-19 pandemic has completely disrupted school day’s structure affecting children’s learning process and health-related behaviours such as physical activity levels, screen exposure, sleep time and eating habits^(3,13–18)^. The relationship to eating habits is of particular concern considering that diet-related risk factors such as high intake of Na, low intake of whole grains and low intake of fruits are the main causes of non-communicable diseases, morbidity and mortality world wide^(19)^.

Ultra-processed foods (UPF) are industrial formulations that are energy-dense; high in added sugars, refined starches, Na and/or unhealthy fats and have little-to-no whole ingredients in their composition^(20)^. These products are convenient, highly palatable, heavily marketed and have a long shelf-life^(20)^. Examples of UPF are flavoured dairy drinks, packaged snacks, prepared frozen dishes, sugary breakfast cereals and salad dressings. A growing body of literature finds an association between increased consumption of UPF with poor diet quality, increased cardiovascular risk factors such as hypertension and increased risk of obesity and non-communicable diseases^(21–23)^. In the past decade, UPF consumption has steadily increased in the paediatric population^(24,25)^. In Chile, a recent study reported that UPF comprised 49 % of Chilean low- and middle-income preschoolers’ total daily energy intake and was therefore, the primary source of energy intake^(26)^. Furthermore, a high proportion of Chilean children consume at least one snack per day (95·2 %)^(27)^, and these snacks are typically energy-dense and high in sugars and saturated fats. Snacks account for nearly 35 % of the calories consumed at school on a given day^(27)^.

Studies documenting eating and lifestyle habits during the COVID-19 pandemic described habits and UPF consumption during lockdown^(15,28,29)^. However, evidence linking UPF consumption to home-school learning environment characteristics during the COVID-19 pandemic is nonexistent. Considering different aspects of the home-school environment, including school adaptation to home-based distance learning models, perceived difficulties for parents due to school closures, as well as variations in child daily routines, could help explain child diets during the COVID-19 pandemic, considering the environment in which children had to eat, play and study. To address these gaps in the literature, this study’s main objective is to assess the association between child UPF consumption and home-school learning environment characteristics during the COVID-19 pandemic in Chile in a sample of elementary schoolchildren from the Food Environment Chilean Cohort (FECHIC). Based on the SDH, we hypothesise that a less structured home-school learning environment is associated with child’s UPF intake during COVID-19. Evidence from this research may inform food environment interventions to support healthier eating habits in children both at home and at school.

## Methods

### Study design and participants

This is a cross-sectional study of schoolchildren from the FECHIC study. The collaboration between the Institute of Nutrition and Food Technology, University of Chile and the National School Assistance and Scholarship Board (JUNAEB) facilitated recruitment of FECHIC participants. The research team collected data of 4–6-year-old children from low- and middle-income neighbourhoods in southeastern Santiago, Chile, in 2016. Because of the young ages of these children, they were invited to participate with their mothers. The study’s inclusion criteria were children from singleton births, without any gastrointestinal disease that could affect their food consumption. Additionally, mothers had to be responsible for household food purchases, had to be the child’s primary caretaker and free of any mental disability or illness. More details about recruitment procedures can be found elsewhere^(26,27)^. In 2020, during the first period of lockdown in Chile, mothers and FECHIC participants were invited to answer an online questionnaire that included nutrition, mental health, among several other biological and psychosocial family aspects. From the 962 children recruited in 2016, 428 children participated in this wave of the study (44·5 %). We compared participants included *v*. those not included in the first wave of the study and found no statistically significant differences by sex, age, nutritional status or mother’s educational level (all p values <0·05). For this study, FECHIC participants were considered eligible if they were still active in 2020 and did not explicitly indicate their voluntary withdrawal from the project; there were no additional exclusion criteria.

### Data collection

#### Student-level data: COVID-19 online survey

The online survey questionnaire was created and managed using REDCap (Research Electronic Data Capture) electronic data capture tools hosted at the Institute of Nutrition and Food Technology at the University of Chile^(30)^. The software generated a unique link for each participant considering their ID. Data used in this study were collected during the COVID-19 pandemic between June and November 2020 (approximately 3–8 months after initial lockdown).

Participants completed two different online surveys: a survey that was designed for adults (adults survey) and was answered by the primary caregiver and a survey that was designed to obtain answers both from caregivers and children (children survey). While the survey was open, participants had an online support service available to resolve any issues. In addition, participants who preferred to answer the survey via telephone or those who left the survey incomplete were given the option to participate in a telephone interview (*n*12, 2·8 %). Participants who completed the survey received a $5000 Chilean pesos electronic gift card in compensation for the time spent with the survey (∼90 min).

Surveys included information about household socio-demographic characteristics, child daily routines, home-school learning environment characteristics, food insecurity, food access, dietary intake, food environment, physical activity, mental health, knowledge and practices related to COVID-19 and food assistance programs. Questionnaires were built using questions validated in other countries^(18,31)^ and in previous studies with the same population^(32)^. For this study, we used survey questions that described home-school learning environment characteristics, child’s diet (UPF consumption) and socio-demographic characteristics that were answered by adults (i.e. in the adult survey, caregivers answered questions about the home-school environment and socio-demographic characteristics. In the child survey, caregivers answered questions regarding the child’s diet).

During the months of data collection, the research team contacted participants up to four times to distribute the survey or to send a reminder to complete it. Each participant was contacted between one to four times, depending on their survey response status.

#### School-level administrative data: National School Assistance and Scholarship Board

School-level administrative data were obtained from the National School Assistance and Scholarship Board (JUNAEB)-Ministry of Education. This organisation conducts annual surveys of anthropometric, socio-demographic and health information of preschool and schoolchildren attending public schools in Chile. Data on geographic location of school, school district (‘comunas^1^’), type of school, eligibility for school meals, school vulnerability index and number of students per school were obtained. Since we had information about the name of the school that each FECHIC participant attended in 2020, we linked the school administrative data from JUNAEB to each school reported by children.

### Outcome

The primary outcome of interest was the mean score of UPF consumption. To assess children’s UPF consumption, we used data collected using the Nova screener, a novel self-report questionnaire that classifies twenty types of UPF according to the Nova system in three food groups: (i) beverages (five subgroups), (ii) products that replace or accompany meals (eight subgroups) and (iii) products often consumed as snacks (seven subgroups)^(33,34)^. The questionnaire asks if the person consumed each of the twenty UPFs listed in the past 24 h and assigns one point if a product of the subgroup was consumed and zero otherwise. The final result is expressed as a Nova score of UPF consumption going from 0 if the child did not consume any of the products to 20 if the child consumed one of each type of the twenty UPF subgroups from the list. For more details, see online Supplementary Table S1. The Nova screener has been validated with Brazilian adults^(33)^, and a recent study showed that the Nova screener was a good predictor of UPF consumption when compared with data on UPF obtained with a 24-h dietary recall in a sample of Chilean preschoolers^(32)^.

### Covariates

To characterise the home-school learning environment, we constructed a home-school scale comprised by three different constructs: school preparedness for online teaching and learning, school closure difficulties for caregivers and daily routine. Questions included were adapted from the University of Oxford Co-SPACE Study^(31)^ and the School Barometer developed by researchers in Germany in the context of the COVID-19 pandemic^(18)^. The first eight questions evaluated parents’ perceptions about teacher and child preparedness for online classes in the pandemic context. The next four questions evaluated parents’ experience with school closures. Lastly, one question evaluated schoolchildren’s routine for daily activities. For more details about the questions and response alternatives, see online Supplementary Table S2.

Socio-demographic variables included child age, child biological sex (male, female, not sure, other, prefer not to say), caregiver mobility (I am leaving home as usual, I am leaving home often but less than before, I am leaving home less, only for essentials, I don’t leave home, don’t know/don’t respond), caregiver employment in the past 2 weeks (student, work at home, employed full-time, employed part-time, unemployed looking, unemployed not looking, don’t know) and mean household income before the pandemic. For further information about covariates, see online Supplementary Table S3.

### Statistical analyses

Statistical analyses were performed using Stata/SE version 17 (College Station, TX, USA). We used descriptive statistics to summarise participants’ characteristics. Mean and standard deviations were used to describe continuous variables (e.g. child Nova score for UPF consumption), and frequencies and percentages were used to describe categorical variables (e.g. caregiver employment in the past 2 weeks). Boxplots and histograms were used to verify implausible values and to evaluate the distribution of each variable included in the study.

#### Construct validity

The statements that characterise each of the constructs (school preparedness for online teaching and learning, school closure difficulties for caregivers and daily routine) were grouped into their associated scales. We constructed three different scales and calculated Cronbach’s-*α* to evaluate the internal consistency of the thirteen questions instrument that captures the three constructs related to the home-school learning environment during the COVID-19 pandemic. Each respondent received a score for each of the scales.

For the first scale (school preparedness for online teaching and learning), respondents were asked to respond to eight statements on a four-point scale, ranging from ‘disagree’ (1) to ‘strongly agree’ (4). ‘I don’t know’ received (0) because it does not contribute information. For statements 1, 2 and 4, we inverted the respondents’ level of agreement to reflect the negative correlation with the options. e.g. 1. Children are not prepared for online learning ranges from ‘strongly agree’ (1) to ‘disagree’ (4).

For the second scale (school closure difficulties), respondents were asked to respond to four statements on a four-point scale, ranging from ‘not difficult at all’ (4) to ‘extremely difficult’ (1). ‘I don’t know’ received (0). The higher the score obtained in this scale, the greater the difficulties faced by parents due to COVID-19 school closures.

For the third scale (daily routine), respondents were asked a question about the child following daily routines for study, play and eating time, with answers ranging from ‘fully’ (4) to ‘not at all’ (1). ‘I don’t know’ received (0). No internal consistency tests were performed for this construct. However, we did assign points considering the ordinal nature of the variable and convert it into an interval variable, essentially assuming that the intervals are equally spaced out. For more information about the scale scoring, see online Supplementary Table S4 and S5.

#### Multiple linear regression analyses

The scores obtained from each of the scales were used in multiple linear regression analyses to determine if home-school learning environment characteristics predict children’s UPF consumption during the COVID-19 pandemic. Models were adjusted for child age, child biological sex, household income, snacking, type of school and eligibility for school meals. Robust standard errors were calculated for all variables. We used two-tailed statistical tests and evaluated significance at 0·1 and 0·05 alpha levels. The model took the following form:

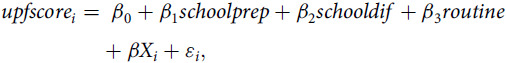

where 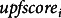 is a continuous measure of the consumption of UPF for child *i*; *β* is the coefficient of the covariates; *β*_1_ is the coefficient of school preparedness for online teaching and learning; *β*_2_ is the coefficient of school difficulties; *β*_3_ is the coefficient of daily routine; *X*_*i*_ is a vector of covariates that includes child age, sex, household income, snacking, type of school and eligibility for school meals and 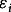 is the error term, assumed to be uncorrelated to all the explanatory variables. Though a Poisson model is recommended to use with outcome variables that are count in nature such as our UPF score variable, we did not observe a Poisson distribution with our data (i.e. Poisson distribution is noticeably skewed) (online Supplementary Fig. S1). Another Poisson model assumption is that the mean of the outcome variable must be equal to its variance, which is not true in our data (mean = 4·3, variance = 3·7). Therefore, we chose to use ordinary least squares regression models.

#### Robustness checks

In addition to the ordinary least squares regression models, we used tobit regression models to account for censoring in the data (i.e. having many observations between 0 and 1). Lastly, we also conducted simple linear regression analyses isolating each construct representing home-school environment as well as adjusting for individual and school-level characteristics.

## Results

### Participant characteristics, ultra-processed food consumption and the home-school learning environment

As described above, 428 surveys were completed between June and November 2020. Most surveys were collected in July 2020 (adult survey: 55·4 %; child survey: 56·3 %).

Table 1 describes participants’ characteristics (*n* 428). Children were on average 8·45 years (sd 0·6) and 53·3 % were female. About a third of households reported a monthly income between $200 000 and $500 000 Chilean pesos (≈ $250 and $625 USD). At the time of the survey, around 85 % of caregivers were leaving home only for essentials errands or not leaving home at all. Work at home was the most common form of employment (51·9 %), followed by caregivers reporting their employment in the past 2 weeks as full-time (20 %).

**Table 1:** Participants’ characteristics, 2020

	*n* 428			
	%	Frequency	Mean	sd
Chile demographic characteristics				
Child age, years			8·45	0·61
Child sex (%)				
Female	53·27	228		
Male	46·73	200		
Household demographic characteristics				
Household reported income in Chilean pesos				
$0–$100 000	3·74	16		
$100 001–$200 000	5·37	23		
$200 001–$300 000	10·98	47		
$300 001–$400 000	12·15	52		
$400 001–$500 000	11·21	48		
$500 001–$600 000	8·88	38		
$600 001–$700 000	7·24	31		
$700 001–$800 000	5·37	23		
$800 001–$900 000	4·44	19		
$900 001–$1000 000	4·44	19		
$1000 001–$1100 000	3·04	13		
$1100 001–$1200 000	2·1	9		
$1200 001–$1500 000	2·8	12		
$1·500·001–$1·800·000	2·34	10		
$1·800·001–$2·000·000	1·64	7		
$2·000·001 and more	4·44	19		
Don’t know/Don’t respond	3·97	17		
Missing	5·84	25		
Caregiver mobility				
I am leaving home as usual	4·67	20		
I am leaving home often but less than before	3·97	17		
I am leaving home less, only for essentials	69·86	299		
I don’t leave home	15·65	67		
Don’t know/ Don’t respond	–	–		
Missing	5·84	25		
Caregiver employment in the past two weeks				
Student	3·5	15		
Work at home	51·87	222		
Employed full-time (40 h/week or more)	20·09	86		
Employed part-time (8–29 h/week)	7·24	31		
Employed part-time (< 8 h/week)	3·04	13		
Unemployed looking for jobs	4·67	20		
Unemployed not looking for jobs	1·4	6		
Don’t know/Don’t respond	2·34	20		
Missing	5·84	25		
Nutrition				
Nova score for UPF consumption			4·25	1·94
Beverage score			2·01	0·94
Food score			1·14	1·05
Snack score			1·1	0·93
Meal structure				
Breakfast	96·73	414		
Mid-morning snack	41·82	179		
Lunch	99·3	425		
Once	81·54	349		
Dinner	23·6	101		
Once-comida	21·26	91		
Snacking				
Yes	83·64	358		
No	16·12	69		
Number of snacking occasions per day			2·14	1·06
Home-school learning environment				
Total home-school score			26·17	8·78
School preparedness for online teaching score			13·36	5·13
School closure difficulties score			10·31	4·13
Daily routine score			2·5	0·93
Child followed a daily routine in the past 2 weeks				
Not at all	12·15	52		
A bit	43·22	185		
A lot	26·17	112		
Completely	18·22	78		
Don’t know	0·23	1		
Daily time spent on schoolwork				
No time	1·4	6		
< 1 h	24·07	103		
1–2 h	31·78	136		
2–3 h	19·39	83		
3–4 h	13·32	57		
5–6 h	3·27	14		
> 6 h	1·17	5		
Don’t know/Don’t respond	1·17	5		
Missing	4·44	19		
Main school activities in the past 2 weeks				
#1 School website	50	214		
#2 Video calls	43·93	188		
#3 Printed study guidelines	35·28	151		
#4 Online learning platform (i.e. Teams)	34·58	148		
#5 School textbooks (printed)	34·35	147		
Household school meals participation before COVID-19				
Yes	44·86	192		
No	55·14	236		

Total home-school score describes the home school learning environment which is composed by three constructs: school preparedness for online teaching (0–24), school closure difficulties (0–16) and daily routine (0–4). Total home-school score goes from 0 to 44 points. Nova score for UPF goes from 0 to 20.

Based on the Nova score, child mean consumption of UPF was 4·3 (sd 1·9) groups, indicating that on average children consumed 4·3 UPF subgroups per day from the twenty UPF subgroups included in the list. Looking at the different UPF categories, children consumed on average 2·0 (sd 0·9) beverages, 1·1 (sd 1·0) products that replace or accompany meals and 1·1 (sd 0·9) snacks in the previous 24 h. Most children reported consuming a snack during the day (83·6 %), and the mean number of snacking occasions per day was 2·1 (sd 1·1). While most children reported to have breakfast (96·7 %), lunch (99·3 %) and ‘once’ (81·5 %) on a given day, only 23·6 % eat at dinner time (Table 1). ‘Once’ is a small late-afternoon meal that is not considered a snack nor is considered dinner. In Chile, ‘once’ sometimes replace dinner and typically includes a cup of tea or coffee, bread and some toppings such as butter, avocado, cheese and ham.

Most children attended large urban public schools in the Santiago Metropolitan area; on average, 80 % of children that attended those schools were eligible to receive school meals (Table 2). Looking at the home-school learning environment during the COVID-19 pandemic, the mean total home-school score was 26·2 (sd 8·8) out of forty-four points. The mean scores for each different construct were 13·4 (sd 5·1) out of 24, 10·3 (sd 4·1) out of 12 and 2·5 (sd 0·9) out of 4 for school preparedness for online teaching and learning, school closure difficulties for caregivers and daily routine, respectively. All scores showed relatively small mean values, suggesting that caregivers did not perceive that schools were prepared for online teaching and learning, experienced difficulties amid school closures, and child daily routines were mostly disrupted. For more detailed description about the frequencies and percentages for responses to questions included in each construct, see online Supplementary Table S3. We found that more than half of children in the sample were not following their daily routine for eating, play and sleep time in the past 2 weeks. In addition, more than half of children in the sample were spending 2 or less hours on schoolwork. Regarding school meals participation, 45 % of households were participating in the school feeding program before the COVID-19 pandemic (Table 1).

**Table 2:** Characteristics of schools attended by participants, 2020

	(*n* 428)			
	%	Frequency	Mean	sd
Demographic Characteristics				
Geographic location				
Urban	92·29	395		
Rural	6·78	29		
Missing	0·93	4		
School district				
La Pintana	13·79	59		
La Florida	6·54	28		
La Granja	7·94	34		
La Cruz	1·64	7		
Llayllay	1·17	5		
Macul	7·01	30		
Ovalle	3·74	16		
Panquehue	2·1	9		
Puente Alto	16·82	72		
Peñalolén	7·24	31		
San Joaquín	1·87	8		
San Miguel	5·61	24		
San Ramón	9·11	39		
Ñuñoa	6·78	29		
Others	8·64	37		
Type of school				
Private	3·97	17		
Public	95·08	407		
Missing	0·93	4		
School size by number of students				
Small	12·38	53		
Midsize	19·86	85		
Large	67·76	290		
Students eligible for school feeding program (%)			78·1	22·68
School vulnerability index-IVE (%)			80·78	11·3

For these analyses, school size measure was defined as: schools with less than 500 students were considered small; schools with 501–1000 students were considered midsize and schools with +1000 students were considered large.

### Construct validity

The performance of the initial construct measuring school preparedness for online teaching and learning (construct 1) with eight questions was marginal (Cronbach-*α* = 0·6494). However, construct 1 exhibited satisfactory internal validity among participants (Cronbach-*α* = 0·7107) after we removed two questions related to home-school learning environment aspects (i.e. ‘We have enough computers/laptops/tablets/smartphones at home’ and ‘We have a comfortable space to study at home’). Thus, the final version of construct 1 reported here included six questions. The construct measuring parents’ experience with school closure difficulties (construct 2) comprised by four questions demonstrated high internal validity among participants (Cronbach-*α* = 0·8483). Correlation item-test, correlation item-rest and Cronbach alpha coefficients for each question are shown in Table 3 and Table 4.

**Table 3:** Home school environment scale validity (internal consistency) I (*n* 401)

Questions	Correlation	Correlation	Cronbach-*α* Coefficient
	Item-test	Item-rest	
Construct 1. School preparedness for online teaching and learning			
1. Children are not prepared for online learning	0·5844	0·398	0·6034
2. Teachers are not prepared for online teaching	0·7179	0·572	0·5549
3. Teachers are motivated to use digital resources for teaching and learning	0·5427	0·3466	0·617
4. Teachers are not prepared to use digital resources for teaching and learning	0·6945	0·5405	0·564
5. School is adequately equipped for online teaching and learning	0·5625	0·3709	0·6106
6. We have enough computers/laptops/tablets/smartphones at home	0·4255	0·2087	0·652
7. We have a comfortable space to study at home	0·2427	0·0107	0·6984
8. Child is motivated with online classes	0·5345	0·3367	0·6196
Total scale			0·6494
Construct 2. School closure difficulties			
9. I find difficult to have a schedule for school activities at home	0·8362	0·692	0·8024
10. I find difficult to maintain communication with teachers	0·7396	0·5455	0·8654
11. I find difficult to supervise child’s schoolwork	0·8912	0·7925	0·7606
12. I find difficult to support child’s learning at home	0·8492	0·7198	0·793
Total scale			0·8483

**Table 4:** Home school environment scale validity (internal consistency) II (*n* 401)

Questions	Correlation item-test	Correlation item-rest	Cronbach-*α* Coefficient
Construct 1. School preparedness for online teaching and learning			
1. Children are not prepared for online learning	0·6607	0·4703	0·6630
2. Teachers are not prepared for online teaching	0·7162	0·5466	0·6485
3. Teachers are motivated to use digital resources for teaching and learning	0·6099	0·4032	0·6837
4. Teachers are not prepared to use digital resources for teaching and learning	0·7300	0·5661	0·6321
5. School is adequately equipped for online teaching and learning	0·6065	0·3988	0·6851
6. Child is motivated with online classes	0·5129	0·2819	0·7195
Total scale			0·7107
Construct 2. School closure difficulties			
7. I find difficult to have a schedule for school activities at home	0·8362	0·6920	0·8024
8. I find difficult to maintain communication with teachers	0·7396	0·5455	0·8654
9. I find difficult to support child’s learning at home	0·8912	0·7925	0·7606
10. I find difficult to supervise child’s schoolwork	0·8492	0·7198	0·7930
Total scale			0·8483

### Association between ultra-processed food consumption and home-school learning environment characteristics

Table 5 shows results from ordinary least squares regression models assessing the association between child UPF consumption and covariates representing home-school learning environment, socio-demographic and school characteristics. We found a statistically significant negative association between child daily routine and child UPF consumption when we adjusted for child socio-demographic (model 1: *β* = –0·19, (95 % CI –0·40, 0·02), *P* value < 0·1) and school characteristics (model 2: *β* = –0·20, (95 % CI –0·41, 0·00), *P*-value < 0·1). These results suggest that for every one-point increase in the daily routine score (suggesting less disruption in child daily routines for eating, play and study during the COVID-19 pandemic), child UPF consumption decreases. Child age was also significantly and inversely associated with child UPF consumption (model 1: *β* = –0·34, (95 % CI –0·65, 0·03, *P* value < 0·05; model 2: *β* = –0·32, (95 % CI –0·63, –0·02), *P* value < 0·05). This finding suggests that for every one-year increase in child’s age, UPF consumption decreases 0·3. In other words, younger children’s UPF consumption was higher than their older counterparts. Associations between child UPF consumption and the other home-school learning environment constructs (i.e. school preparedness for online teaching and learning and school closure difficulties for caregivers) were NS.

**Table 5:** Multiple linear regression models predicting UPF consumption

Variable	Model 1 *β*	95 % CI	Model 2 *β*	95 % CI
Home-school learning environment				
School preparedness	0·01	–0·04, 0·07	0·01	–0·04, 0·06
School closure diff	−0·00	–0·06, 0·06	−0·00	–0·06, 0·06
Daily routine	−0·19*	–0·40, –0·02	−0·20*	–0·41, 0·00
Age (years)	−0·34**	–0·65, 0·03	−0·32**	–0·63, –0·02
Female	0·11	–0·27, 0·48	0·08	–0·29, 0·46
Snacking	0·21	–0·29, 0·72	0·22	–0·29, 0·72
Public school			0·43	–0·92, 1·78
Eligibility for school meals			−0·00	–0·01, 0·01
Constant	7·18**	4·46, 9·91	6·89**	3·90, 9·87
Observations	426		422	
F statistic	1·42		1·11	
Prob > F	0·1939		0·3516	

* *P* < 0·1.

** *P* < 0·05. Total home-school score describes the home school learning environment which is composed by three constructs: school preparedness for online teaching (0–24), school closure difficulties (0–16) and daily routine (0–4). Nova score for UPF goes from 0 to 20.

### Robustness checks

Results from the tobit regression models were consistent with the ordinary least squares models previously described (online Supplementary Table S6). In the simple linear regression models (online Supplementary Table S7), we found no association between the school preparedness for online teaching and learning and child UPF consumption (model 6). The school closure difficulties construct was not associated with the outcome either (model 7). However, we found a statistically significant association between daily routine and child UPF consumption both when daily routine was introduced in the model alone as well as in the presence of the other two constructs (model 8: *β* = –0·18, (95 % CI –0·02, 0·02), *P* value < 0·1; model 9: *β* = –0·19, (95 % CI –0·39, 0·02), *P* value < 0·1). In online Supplementary Table S7 and S8, we observe that the association between daily routine and UPF remained consistent when we included school-level variables and other child demographic characteristics (Model 10–13).

## Discussion

Our study using data from a sample of schoolchildren in Santiago, Chile, shows that variations in child daily routine to study, eat and play during the COVID-19 pandemic were negatively associated with UPF intake. Further, we did not find associations between other home-school learning environment constructs and UPF intake. To our knowledge, this is the first study to examine associations between home-school learning environment characteristics during the COVID-19 pandemic and UPF consumption in low- and middle-income schoolchildren. These results offer insight into how substantial differences in child daily routine may have an impact on health outcomes (i.e. UPF intake) associated with obesity and non-communicable diseases.

Our results suggest that less structured daily routines during this period of home schooling promote unhealthier behaviours in schoolchildren. The structured days hypothesis postulates that the protective elements of the structured school day related to diets are the limited (and scheduled) opportunities children have to eat during their time at school (i.e. breakfast, lunch, break) and the regulated access to healthier food and beverages as well as to nutritious school meals^(10)^. During the COVID-19 pandemic, children spent most of their time at home, an environment in which there is less structure, regulation and routine. Based on the ‘filled-time perspective’, the school environment contributes to fill schoolchildren’s time with favourable activities such as structured mealtimes at school and leaves less time to unfavourable activities such as snacking on unhealthier food and beverages, long periods of screen time and sedentary behaviours at home^(10)^. Several studies have documented that strict home confinement and school closures during the COVID-19 pandemic promoted sedentary behaviours such as an increase in screen time, a decrease in physical activity levels, changes in children’s emotional state, less time sleeping and increased snacking on energy-dense nutrient poor foods and beverages^(7,13–16,28,35,36)^. All these behaviours could have been a result of daily routine disruptions, and they might co-occur and lead to an increased intake of UPF. Although other explanations are possible, we argue that the structured days hypothesis provides a strong case that can be applied to any scenario that disrupted child structured school days such as the COVID-19 pandemic.

Though our study did not evaluate changes in UPF intake before and after lockdown, our results suggest that variations in child daily routine are negatively associated with UPF intake. Our findings are in line with a multi-country study that evaluated youth UPF intake during the COVID-19 pandemic^(15)^. Authors found an increased habitual consumption of UPF, especially in Latin American countries^(15)^. Another study conducted in Chile showed that the percentage of compliance with sweet (i.e. cookies, chocolates or sweets) or salty (i.e. potato chips or ultra-processed corn tortillas flavoured with cheese, ham or similar) snacks intake was only 27 % for preschoolers and 24 % for adolescents^(14)^. There are several reasons why UPF intake might have increased during home confinement and school closures. First, due to the uncertainty when the COVID-19 pandemic started and to minimise trips to the grocery store, households started to buy and stockpile more foods with a longer shelf-life including ultra-processed snacks, canned and frozen food that are high in energy content, sugar, Na and unhealthy fats^(37)^. Along these lines, UPF are known by their convenience and practicality because they mostly come in ready-to-eat packages and require little to no preparation time^(20)^. In addition, caregivers’ difficulties with balancing working from home and family life might have caused stress and other psychological issues making caregivers choose the easiest and more convenient option to feed their children^(35)^. Lastly, children were more sedentary and more exposed to screens during lockdown^(17,36,38)^; therefore, they were more susceptible to the influence of advertisements of UPF, which were prevalent during lockdown^(39,40)^. Although in Chile this effect might have been tempered by the application of the Chilean Food Labeling and Advertising Law^(41)^.

Examining the home-school learning environment during the COVID-19 pandemic in our sample, we observe an environment where children spent less time than usual doing schoolwork, barely following a daily routine and struggling to adjust to online learning. Concerningly, more than half of children in our sample spent 2 h or less on schoolwork. A possible explanation for this finding might be related to inequalities in learning resources available at home and those provided by schools to transition to online education^(2,6)^. A survey conducted in September 2020 as part of the longitudinal study ‘Empleo COVID-19’ with 16·000 households in Chile reported that 65·5 % of schoolchildren received classes through video calls, 24·1 % through printed study guides and 20·6 % through school texts^(41)^. Zooming in and looking at these data by income quintiles, authors reported that online classes were less prevalent in lower quintiles (60·6 %) compared to higher quintiles (84·3 %)^(42)^. A difference was also observed across type of school where around 90 % of school children that attended private schools received online classes *v*. only 61·2 % that attended public schools^(42)^. Conversely, the authors found a greater prevalence of classes provided via printed study guides (Q1 30·3 % *v*. Q5 5·5 %) and school texts (Q1 22·1 % *v*. Q5 10·2 %) in lower quintiles^(42)^. In our study, we found a lower prevalence of online classes (44 %) but similar results regarding classes with study guides (35 %) and school texts (34 %). Additionally, in our study, 42 % of the study sample disagreed with the statement ‘We have enough computers/laptops/tablets/smartphones at home’, making it difficult to adapt to online classes. This finding may be related to the low- and middle-income status of children in our sample and demonstrates the gap that exists across income levels concerning teaching and learning resources both at home and at school during the COVID-19 pandemic^(6)^.

Another interesting finding to discuss is the association between child age and UPF consumption. We found that older children consumed less UPF than their younger counterparts. Other studies have reported higher UPF intake in young children compared with older children^(24,43)^. In the context of the COVID-19 pandemic, this finding may also be explained by the higher level of COVID-related parental stress that could have led to the use of UPF snacks for emotional feeding in younger children that demand more attention and care^(34)^. Considering the multiple responsibilities of parents and the challenge to balance work, childcare and home schooling during the pandemic, stressed parents may use coercive and coping strategies to manage children’s behaviours^(44)^. Jansen and colleagues documented an association between COVID-19-related parental stress and child UPF snacks intake^(35)^. Conversely, Wang and colleagues analysed trends in UPF consumption among youth in the USA and found that older youth (i.e. aged 6–11 and aged 12–19 years) had a significantly higher UPF intake compared with those aged 2–5 years^(25)^. Authors suggest that this result is related to the higher exposure to marketing, availability and choice of UPF for older youth. In Chile, however, the Chilean Law of Food Labeling and Advertising bans all forms of marketing directed to children. Nevertheless, further research is needed to understand how media was compliant with Chile’s Law during home confinement and school closures, and how this could have impacted younger children’s UPF intake.

It is likely that the homogeneity of the study sample and of schools that participants attended played a role in the non-significant associations between the two constructs that were more related to the school learning environment and UPF intake. The same could have happened with associations between type of school or eligibility for school meals and UPF intake. Most children in our sample were from low- and middle-income households in southeastern Santiago and attended mostly urban public schools with a high percentage of kids eligible for school meals. Further research is needed to understand if these non-significant associations remain in a more heterogeneous sample across type of school and educational levels.

### Strengths and limitations

The main strengths of this study include the use of a novel tool with validated questions^(18,31)^ to assess three different constructs to characterise the learning environment during home confinement and school closures.

We acknowledge our study also has limitations. First, this is a cross-sectional study, and we only analysed the first wave of data collected during the COVID-19 pandemic in Santiago, Chile. Therefore, it only gives us information about what happened in a specific point in time during the COVID-19 pandemic and causality cannot be attributed. Our survey was conducted over a 5-month period, and the effects of confinement might have varied over that period; however, more than 75 % of the responses were collected between July and August, decreasing this potential differential impact. In addition, we cannot ignore that there may be underlying behavioural characteristics that lead to children who are more organised in their routines also being more organised in how they eat (i.e. it is not that routines impact dietary behaviours but rather some psychological traits explain both types of behaviours). There is a need for longitudinal studies that evaluate what happened in this study cohort during other stages of the COVID-19 pandemic and after strict home confinement measures were lifted and children return to schools for in-person classes. Second, the sample was composed of low- and middle-income children living in urban southeastern Santiago, and findings may not be generalisable to other areas of Chile that could have had different experiences with home confinement and school closures during the COVID-19 pandemic. Third, UPF consumption was assessed using self-reported intake in the previous 24 h, and this method is subject to social desirability bias, which may lead to lower reported intakes of UPF. In addition, the Nova score is a diversity measure rather than an absolute intake measure, which limits the ability to evaluate the quantity of energy content from UPF consumed as well as to estimate nutrient intake. Lastly, participants are part of a nutrition cohort that could have influenced their survey responses. Nevertheless, the novel simplified tool we used to assess UPF (Nova score) has been validated in other populations and has shown good agreement with data reported through 24-h dietary recalls by participants of the same study cohort^(32)^. Lastly, we constructed a scoring scale to assess different constructs associated with the home-school learning environment during home confinement and school closures. Yet, the score assignment was *ad hoc*, and this may influence results interpretation.

### Conclusions

We found that variations in child daily routine to eat, play and study were negatively associated with UPF intake in a sample of schoolchildren from low- and middle-income neighbourhoods in Chile. Disruptions in child daily routine led to a less structured day which has been associated with unhealthy behaviours. The school day structure and a regulated school environment can provide schoolchildren with a healthier environment and contribute to improving child diets. Future research is needed to understand if and how child UPF intake and daily routine relate when schools reopen.

## Footnotes

1
Comunas are decentralized political and administrative management units of the City. Each comuna has its own legal status. For instance, Santiago is composed of 40 comunas.

## Supplementary material

For supplementary material accompanying this paper visit https://doi.org/10.1017/S1368980023001593. click here to view supplementary material
