# Supplementary material for: Ultra-processed food consumption is associated with variations in daily routines in elementary schoolchildren during the COVID-19 pandemic in Chile
Source: Public Health Nutr. 2023 Aug 2;26(10):1956–67. doi: 10.1017/S1368980023001593 (PMC10564603; doi:10.1017/S1368980023001593)
Supplement: Supplementary file 1 [file S1368980023001593sup001.docx]

**SUPPLEMENTARY MATERIAL**

**Table S1. Food screener for ultra-processed foods**

| **Food group** | **Question** | **Alternatives** |
| --- | --- | --- |
| Beverages | Which of the following beverages did you drink yesterday? Choose all that apply. | 1. Regular or diet soda  2. Canned or bottled fruit juice  3.Chocolate milk (canned or powder)  4. Tea or coffee-based drink  5. Flavored yogurt  6. None of the above  7. Don’t know |
| Products that replace or accompany meals | Which of the following foods did you eat yesterday? Choose all that apply. | 1. Sausage, hamburgers or nuggets  2. Ham, mortadella or salami  3. Loaf bread or other type of packaged bread  4. French fries from store or restaurant  5. Mayonnaise, ketchup, or margarine  6. Instant noodles, packaged soup or other ready-to-eat foods  7. Frozen lasagna or other frozen ready-made meals  8. Salad dressing  9. None of the above  10. Don’t know |
| Products often consumed as snacks | Which of the following snacks did you eat yesterday? Choose all that apply. | 1. Potato chips or any kind of salty snacks  2. Packaged cookies, biscuits or similar  3. Packaged cakes, muffins or pies  4. Cereal bar  5. Ice cream or frozen desserts  6. Chocolate bar or candy with chocolate  7. Packaged sugary breakfast cereal  8. None of the above  9. Don’t know |

**Table S2. Home-school learning environment assessment tool**

| **Construct** | **Questions** | **Alternatives** |
| --- | --- | --- |
| School preparedness for online teaching and learning | 1. Children are not prepared for online learning  2. Teachers are not prepared for online teaching  3. Teachers are motivated to use digital resources for teaching and learning  4. Teachers are not prepared to use digital resources for teaching and learning  5. School is adequately equipped for online teaching and learning  6. We have enough computers/laptops/tablets/smartphones at home  7. We have a comfortable space to study at home  8. Child is motivated with online classes | Disagree  Somewhat agree  Agree  Strongly agree  Don’t know |
| School closure difficulties | 1. How difficult is to have a schedule for school activities at home  2. How difficult is to maintain communication with teachers  3. How difficult is to supervise child’s schoolwork  4. How difficult is to support child’s learning at home | Not difficult at all  Somewhat difficult  Very difficult  Extremely difficult  Don’t know |
| Daily routine | 1. In the past two weeks, has the child followed a routine for daily activities? (e.g., study time, play time, eating time) | Not at all  A little  Pretty much  Fully  Don’t know |

**Table S3. Covariates**

| **Variable** | **Question** | **Alternatives** |
| --- | --- | --- |
| *Demographics*  Child age  Child sex      Caregiver employment situation    Caregiver social isolation status      Mean household income | How old are you?  You are…  What was your main activity in the past two weeks?  What is your current isolation status? Select only one option.  What was the mean household income before the COVID-19 pandemic? | Continuous  Categorical: Male, female, not sure, other, prefer not to say  Categorical: Student, housework, employed full-time, employed part-time, unemployed looking, unemployed not looking, don’t know  Categorical: I am leaving home as usual, I am leaving home often but less than before, I am leaving home less, only for essentials, I don’t leave home, don’t know/don’t respond  Categorical: 16 categories |
| *School*  Household school meals participation    Schoolwork  Main school activities | Usually, before the COVID-19 pandemic, were you or someone in your family beneficiary of the following programs?  In the past two weeks, how much time per day the child spent doing schoolwork?  Choose 5 school activities that the child has done in the past two weeks | Categorical: yes, no  Categorical: no time, <1 hour, 1-2 hours, 2-3 hours, 3-4 hours, 5-6 hours, >6 hours, don’t know  Categorical (14 options. E.g., homework via email, homework via text message, homework downloaded from website…) |
| *Eating structure*  Meal structure      Snacking    Number of snacking  occasions per day | Which meals does the child eat most days? Choose all that apply  Does the child eat between meals most days? (e.g., cookies, bread, fruit, yogurt, soda)  How many times a day? | Categorical: Breakfast, mid-morning snack, lunch, once, dinner, once-comida  Categorical: Yes, no, don’t know  Continuous |

**Table S4. The thirteen questions included in the survey grouped into the three constructs with the corresponding scale scoring**

| **Construct 1. School preparedness for online teaching and learning** | **Disagree** | **Somewhat agree** | **Agree** | **Strongly agree** | **DK/DR** | **Missing** |
| --- | --- | --- | --- | --- | --- | --- |
| 1. Children are not prepared for online learning  2. Teachers are not prepared for online teaching  3. Teachers are motivated to use digital resources for teaching and learning  4. Teachers are not prepared to use digital resources for teaching and learning  5. School is adequately equipped for online teaching and learning  6. We have enough computers/laptops/tablets/smartphones at home  7. We have a comfortable space to study at home  8. Child is motivated with online classes | 4  4  1  4  1  1  1  1 | 3  3  2  3  2  2  2  2 | 2  2  3  2  3  3  3  3 | 1  1  4  1  4  4  4  4 | 0  0  0  0  0  0  0  0 | 0  0  0  0  0  0  0  0 |
| **Construct 2. School closure difficulties** | **Not difficult at all** | **Somewhat difficult** | **Very difficult** | **Extremely difficult** | **DK/DR** | **Missing** |
| 1. I find difficult to have a schedule for school activities at home 2. I find difficult to maintain communication with teachers 3. I find difficult to supervise child’s schoolwork 4. I find difficult to support child’s learning at home | 4  4  4  4 | 3  3  3  3 | 2  2  2  2 | 1  1  1  1 | 0  0  0  0 | 0  0  0  0 |
| **Construct 3. Daily routine** | **Not at all** | **A bit** | **A lot** | **Completely** | **DK/DR** | **Missing** |
| 13. In the past two weeks, has the child followed a routine for daily activities? (e.g., study time, play time, eating time) | 1 | 2 | 3 | 4 | 0 | 0 |

DK/DR=don’t know/don’t respond

**Table S5. Distribution of home school environment characteristics by construct (n=428)**

| **Constructs** | **%**  **(n)** | | | | | |
| --- | --- | --- | --- | --- | --- | --- |
| **Construct 1. School preparedness for online teaching and learning** | **Disagree** | **Somewhat agree** | **Agree** | **Strongly agree** | **DK/DR** | **Missing** |
| 1. Children are not prepared for online learning  2. Teachers are not prepared for online teaching  3. Teachers are motivated to use digital resources for teaching and learning  4. Teachers are not prepared to use digital resources for teaching and learning  5. School is adequately equipped for online teaching and learning  6. We have enough computers/laptops/tablets/smartphones at home  7. We have a comfortable space to study at home  8. Child is motivated with online classes | 12.15  (52)  18.22  (78)  5.61  (24)  28.27  (121)  23.83  (102)  42.06  (180)  24.77  (106)  32.01  (137) | 34.81  (149)  39.25  (168)  33.41  (143)  40.42  (173)  38.08  (163)  24.53  (105)  37.15  (159)  42.52  (182) | 24.07  (103)  19.86  (85)  30.61  (131)  11.45  (49)  19.16  (82)  13.08  (56)  18.22  (78)  13.55  (58) | 21.73  (93)  14.49  (62)  18.93  (81)  7.24  (31)  7.01  (30)  13.08  (56)  13.32  (57)  5.14  (22) | 0.93  (4)  1.87  (8)  5.14  (22)  6.31  (27)  5.61  (24)  0.93  (4)  0.23  (1)  0.47  (2) | 6.31  (27)  6.31  (27)  6.31  (27)  6.31  (27)  6.31  (27)  6.31  (27)  6.31  (27)  6.31  (27) |
| **Construct 2. School closure difficulties** | **Not difficult at all** | **Somewhat difficult** | **Very difficult** | **Extremely difficult** | **DK/DR** | **Missing** |
| 1. I find difficult to have a schedule for school activities at home 2. I find difficult to maintain communication with teachers 3. I find difficult to supervise child’s schoolwork 4. I find difficult to support child’s learning at home | 19.39  (83)  37.85  (162)  18.46  (79)  20.79  (89) | 38.08  (163)  34.35  (147)  35.28  (151)  42.99  (184) | 17.06  (73)  12.62  (54)  20.56  (88)  17.29  (74) | 18.93  (81)  7.94  (34)  19.16  (82)  12.38  (53) | 0.23  (1)  0.93  (4)  0.23  (1)  0.23  (1) | 6.31  (27)  6.31  (27)  6.31  (27)  6.31  (27) |
| **Construct 3. Daily routine** | **Not at all** | **A bit** | **A lot** | **Completely** | **DK/DR** | **Missing** |
| 13. In the past two weeks, has the child followed a routine for daily activities? (e.g., study time, play time, eating time) | 12.15  (52) | 43.22  (185) | 26.17  (112) | 18.22  (78) | 0.23  (1) | - |

DK/DR=don’t know/don’t respond; frequencies are in parenthesis

**Robustness checks**

**Table S6. Tobit regression models predicting UPF consumption**

| **Variable** | **Model 3**  $\boldsymbol{\beta}$  **(95% CI)** | **Model 4**  $\boldsymbol{\beta}$  **(95% CI)** |
| --- | --- | --- |
| *Home-school learning environment*  School preparedness  School closure diff  Daily routine | 0.01  (-0.04 – 0.06)  -0.00  (-0.06 – 0.06)  -0.19*  (-0.40– 0.01) | 0.01  (-0.04 – 0.06)  -0.00  (-0.06 – 0.06)  -0.20*  (-0.41 – 0.00) |
| Age (years) | -0.34**  (-0.64 – 0.03) | -0.31**  (-0.63 – -0.00) |
| Female | 0.12  (-0.25 – 0.49) | 0.09  (-0.28 – 0.47) |
| Snacking | 0.22  (-0.28 – 0.72) | 0.22  (-0.28 – 0.73) |
| Public school |  | 0.44  (-0.89 – 1.77) |
| Eligibility for school meals |  | -0.00  (-0.01 – 0.01) |
| Constant | 7.16**  (4.45 – 9.87) | 6.88**  (3.91 – 9.85) |
| Observations | 426 | 422 |
| Log likelihood | -888.12 | -874.89 |
| Prob > chi2 | 0.1880 | 0.3273 |

*p<0.1; **p<0.05. Total home-school score describes the home school learning environment which is composed by three constructs: school preparedness for online teaching (0-24), school closure difficulties (0-16) and daily routine (0-4). Nova score for UPF goes from 0 to 20.

Model 3 and 4 with left-censoring ll(0).

**
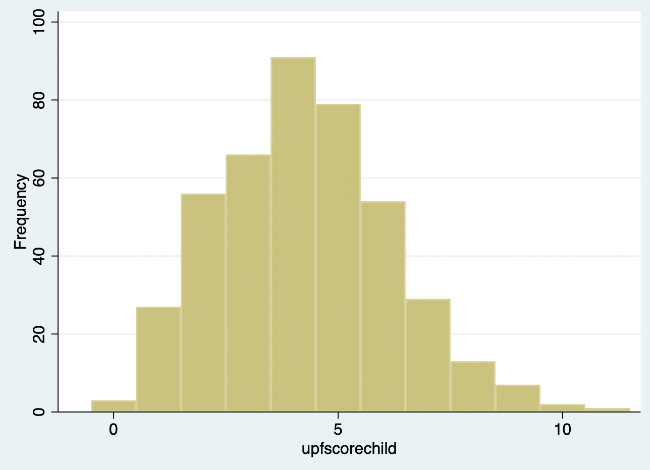
**
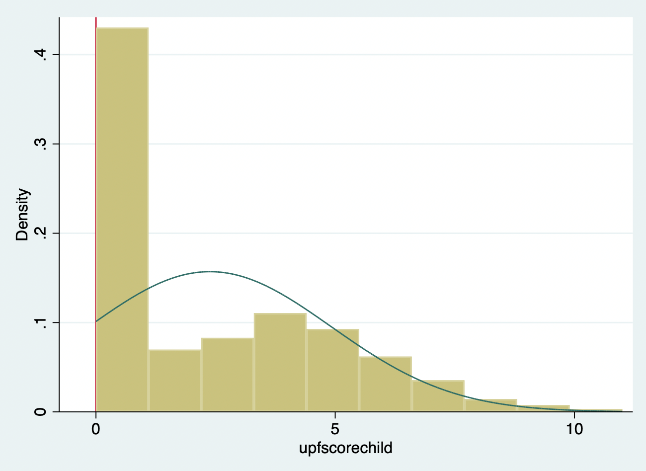
**Figure S1. Child UPF intake distribution**

**Table S7. Simple and multiple linear regression models predicting UPF consumption**

| **Variable** | **Model 5**  **B (95% CI)** | **Model 6**  **B (95% CI)** | **Model 7**  **B (95% CI)** | **Model 8**  **B (95% CI)** | **Model 9**  **B (95% CI)** | **Model 10**  **B (95% CI)** | **Model 11**  **B (95% CI)** |
| --- | --- | --- | --- | --- | --- | --- | --- |
| *Home-school learning environment*  Total home-school score  School preparedness  School closure diff  Daily routine | -0.00  (-0.02 – 0.02) | 0.01  (-0.03 – 0.04) | -0.00  (-0.05 – 0.04) | -0.18*  (-0.02 – 0.02) | 0.02  (-0.03 – 0.06)  -0.01  (-0.06 – 0.06)  -0.19*  (-0.39 – 0.02) | 0.01  (-0.04 – 0.06)  -0.01  (-0.07 – 0.06)  -0.20*  (-0.40 – 0.01) | 0.01  (-0.04 – 0.06)  -0.01  (-0.07 – 0.06)  -0.20*  (-0.40 – 0.01) |
| Public school |  |  |  |  |  | 0.30  (-0.65 – 1.25) |  |
| Eligibility school meals |  |  |  |  |  |  | 0.00  (-0.01 – 0.01) |
| Constant | 4.28**  (-0.02 – 0.02) | 4.18**  (3.67 – 4.70) | 4.28**  (3.79 – 4.79) | 4.70**  (4.17 – 5.23) | 4.58**  (3.92 – 5.25) | 4.35***  (3.17 – 5.53) | 4.65***  (3.66 – 5.63) |
| Observations | 428 | 428 | 428 | 428 | 428 | 424 | 424 |
| F statistic | 0.01 | 0.08 | 0.02 | 3.15 | 1.20 | 1.07 | 0.97 |
| Prob > F | 0.9254 | 0.7795 | 0.8835 | 0.0765 | 0.3101 | 0.3715 | 0.4227 |

*p<0.1; **p<0.05. Total home-school score describes the home school learning environment which is composed by three constructs: school preparedness for online teaching (0-24), school closure difficulties (0-16) and daily routine (0-4). Nova score for UPF goes from 0 to 20.

**Table S8. Additional multiple linear regression models predicting UPF consumption**

| **Variable** | **Model 12**  **B (95% CI)** | **Model 13**  **B (95% CI)** |
| --- | --- | --- |
| *Home-school learning environment*  Total home-school score  School preparedness  School closure diff  Daily routine | 0.02 (-0.03– 0.07)  -0.01 (-0.07 – 0.06)  -0.21* (-0.42– 0.00) | 0.02 (-0.04 – 0.07)  -0.00 (-0.07 – 0.07)  -0.23* (-0.46 – 0.00) |
| Household income  $0 - $100,000  $100,001 - $200,000  $200,001 - $300,000  $300,001 - $400,000  $400,001 - $500,000  $500,001 - $600,000  $600,001 - $700,000  $700,001 - $800,000  $800,001 - $900,000  $900,001 - $1,000,000  $1,000,001 -$ 1,100,000  $1,100,001 - $1,200,000  $1,200,001 - $1,500,000  $1,500,001 - $1,800,000  $1,800,001 - $2,000,000  $2,000,001 and more |  | Reference  -0.73 (-2.09 – 0.63)  -0.33 (-1.56 – 0.90)  -1.07 (-2.30 – 0.15)  -0.81 (-2.05 – 0.42)  -0.53 (-1.80 – 0.75)  -0.59 (-1.89 – 0.71)  -0.04 (-1.41 – 1.32)  -1.16 (-2.56 – 0.24)  -0.31 (-1.72 – 1.08)  0.08 (-1.47 – 1.63)  0.00 (-1.69 – 1.70)  -0.79 (-2.35 – 0.78)  -0.81 (-2.46 – 0.83)  -1.40 (-3.23 – 0.44)  -0.62 (-2.06 – 0.81) |
| Age (years) | -0.37** (-0.68 – 0.05) | -0.31* (-0.65 – 0.03) |
| Female | 0.11 (-0.27 – 0.49) | 0.12 (-0.29 – 0.52) |
| Snacking | 0.25 (-0.26 – 0.77) | 0.22 (-0.34 – 0.77) |
| Daily time spent on schoolwork  0-2 hours  2-4 hours  5 or more hours | Reference  0.05 (-0.36 – 0.47)  -0.53 (-1.45 – 0.39) | Reference  -0.01 (-0.46 – 0.44)  -0.64 (-1.60 – 0.33) |
| Constant | 7.48** (4.71 – 10.26) | 7.65** (4.38 – 10.92) |
| Observations | 408 | 385 |
| F statistic | 1.32 | 0.89 |
| Prob > F | 0.2162 | 0.6208 |

*p<0.1; **p<0.05

**Table S9. Consumption frequency (%) of foods and beverages included in the Nova screener for the consumption of UPF in schoolchildren in Chile, 2020 (n=428)**

| **Food group** | **Foods and beverages** | **% (n)** |
| --- | --- | --- |
| Beverages | 1. Regular or diet soda  2. Canned or bottled fruit juice  3.Chocolate milk (canned or powder)  4. Tea or coffee-based drink  5. Flavored yogurt  6. None of the above  7. Don’t know/don’t respond | 15.4 (66)  20.3 (87)  7.2 (31)  36.9 (158)  8.6 (37)  3.9 (17)  0.5 (2) |
| Products that replace or accompany meals | 1. Sausage, hamburgers or nuggets  2. Ham, mortadella or salami  3. Loaf bread or other type of packaged bread  4. French fries from store or restaurant  5. Mayonnaise, ketchup, or margarine  6. Instant noodles, packaged soup or other ready-to-eat foods  7. Frozen lasagna or other frozen ready-made meals  8. Salad dressing  9. None of the above  10. Don’t know/don’t respond | 6.3 (27)  14.3 (61)  16.6 (71)  2.0 (8)  11.2 (48)  8.8 (37)  0.2 (1)  1.6 (7)  17.1 (73)  - |
| Products often consumed as snacks | 1. Potato chips or any kind of salty snacks  2. Packaged cookies, biscuits or similar  3. Packaged cakes, muffins or pies  4. Cereal bar  5. Ice cream or frozen desserts  6. Chocolate bar or candy with chocolate  7. Packaged sugary breakfast cereal  8. None of the above  9. Don’t know/don’t respond | 6.5 (28)  12.0 (51)  5.1 (22)  1.6 (7)  4.7 (20)  4.4 (19)  6.1 (26)  21.73 (93)  0.5 (2) |

**Table S10. UPF intake frequency by score (n=428)**

| **Nova score for the consumption of UPF** | **% (n)** |
| --- | --- |
| 0-1 | 7.0 (30) |
| 2 | 13.1 (56) |
| 3 | 15.4 (66) |
| 4 | 21.3 (91) |
| 5 or more | 43.2 (185) |

**Table S11. Multiple linear regression models predicting UPF consumption (n=428) with dummy variables for daily routine categories**

| **Variable** | $\boldsymbol{\beta}$  **(95% CI)** | $\boldsymbol{\beta}$  **(95% CI)** | $\boldsymbol{\beta}$  **(95% CI)** |
| --- | --- | --- | --- |
| *Home-school learning environment*  Total home-school score  School preparedness  School closure diff  Daily routine  Not at all  A bit    A lot  Completely | Reference  -0.24  (-0.83 – 0.36)  -0.51  (-1.15 – 0.12)  -0.51  (-1.19 – 0.17) | 0.01  (-0.04 – 0.06)  -0.00  (-0.06 – 0.06)  Reference  -0.21  (-0.81– 0.39)  -0.52  (-1.17 – 0.12)  -0.51  (-1.21 – 0.19) | 0.01  (-0.04 – 0.06)  -0.00  (-0.06 – 0.06)  Reference  -0.22  (-0.84 – 0.39)  -0.56*  (-1.23 – 0.09)  -0.54  (-1.25 – 0.17) |
| Age (years) |  | -0.34**  (-0.65 – 0.03) | -0.32**  (-0.64 – -0.01) |
| Female |  | 0.11  (-0.26 – 0.49) | 0.09  (-0.29 – 0.47) |
| Snacking |  | 0.20  (-0.31 – 0.71) | 0.20  (-0.31 – 0.71) |
| Public school |  |  | 0.41  (-0.97 – 1.77) |
| Eligibility for school meals |  |  | -0.00  (-0.01 – 0.01) |
| Constant | 4.58**  (4.06 – 5.11) | 7.11**  (4.38 – 9.84) | 6.85**  (3.84 – 9.86) |
| Observations | 428 | 426 | 422 |
| F statistic | 1.20 | 1.16 | 0.97 |
| Prob > F | 0.3106 | 0.3174 | 0.4722 |

*p<0.1; **p<0.05. Total home-school score describes the home school learning environment which is composed by three constructs: school preparedness for online teaching (0-24), school closure difficulties (0-16) and daily routine (0-4). Nova score for UPF goes from 0 to 20.

**Table S12. Frequency of survey responses by month (n=428)**

|  | **Adult survey** | **Child survey** |
| --- | --- | --- |
| **Month, 2020** | **% (n)** | **% (n)** |
| June | 15.4 (66) | 0 |
| July | 55.4 (237) | 56.3 (241) |
| August | 17.1 (73) | 34.6 (148) |
| September | 7.7 (33) | 8.2 (35) |
| October | 0.5 (2) | 0.9 (4) |
| Missing | 4.0 (17) | - |
